# Post-cardiac surgery mortality in ICU patients with serum glucose–potassium ratio: A retrospective cohort analysis of the MIMIC-IV database

**DOI:** 10.1097/MD.0000000000049469

**Published:** 2026-06-19

**Authors:** Yan Wu, Dandan Xu, Jun Lu, Lin Zhang, Zhonglan Cai, Guang Tu

**Affiliations:** a Nursing Department, Shanghai Public Health Clinical Center (Fudan University), Shanghai, China; b The Second Department of Interior, Lichuan County People’s Hospital of Jiangxi Province, Fuzhou, China.

**Keywords:** cardiac surgery, glucose–potassium ratio (GPR), in-hospital mortality, intensive care unit (ICU), MIMIC-IV database, prognostic marker

## Abstract

The serum glucose–potassium ratio (GPR) has emerged as a potential marker for various clinical outcomes. However, its association with post-cardiac surgery mortality in intensive care unit (ICU) patients remains unclear. To evaluate the association between GPR and in-hospital mortality in patients following cardiac surgery admitted to the ICU. This retrospective cohort study analyzed data from the Medical Information Mart for Intensive Care, version IV database (2008–2019). We included 590 patients aged 71.4 years on average, with a majority being male (66.4%) and non-White (69.8%). The primary exposure was GPR, defined as the ratio of serum glucose to potassium levels within 24 hours of ICU admission. Cox proportional hazards models were used to assess the relationship between GPR and in-hospital mortality, with adjustments for confounding variables. The univariate Cox regression analysis showed a significant association between GPR and in-hospital mortality (hazard ratio: 1.06; 95% confidence interval: 1.03–1.08, *P* < .001). This relationship persisted in multivariate models (Model 3: hazard ratio: 1.07; 95% confidence interval: 1.04–1.11, *P* < .001). Quartile analysis indicated that patients in the highest quartile of GPR (Q4 > 33) had a significantly higher risk of mortality compared to those in the lowest quartile (Q1 < 26). Subgroup analyses revealed significant interactions between GPR and age, with a trend towards higher mortality risk in older patients. In this cohort of post-cardiac surgery ICU patients, higher GPR was associated with increased in-hospital mortality. Further research is needed to explore the clinical implications of GPR as a potential prognostic marker in this patient population.

## 1. Introduction

Cardiac surgery is a common and often life-saving procedure that can lead to significant physiological stress, impacting patient outcomes.^[1]^ Postoperative care in the intensive care unit (ICU) is crucial for monitoring and managing complications that can arise following cardiac surgery.^[2]^ Despite advancements in surgical techniques and critical care management, in-hospital mortality remains a significant concern, highlighting the need for better risk stratification tools.^[3]^ The serum glucose–potassium ratio (GPR) has been identified as a potential biomarker in various clinical settings, including cardiovascular diseases.^[4]^ GPR is calculated by dividing serum glucose levels by serum potassium levels and has been associated with inflammation, oxidative stress, and metabolic derangements.^[5]^ These factors are particularly relevant in the post-cardiac surgery population, where metabolic disturbances are common and can impact clinical outcomes.^[6]^ While several studies have explored the prognostic value of GPR in different patient populations,^[7–11]^ its role in predicting in-hospital mortality following cardiac surgery remains understudied.

The Medical Information Mart for Intensive Care, version IV (MIMIC-IV) database, a large publicly available dataset of ICU patients, provides an opportunity to investigate the association between GPR and clinical outcomes in a well-defined cohort of post-cardiac surgery patients.^[12]^ This study aims to evaluate the association between GPR and in-hospital mortality in patients admitted to the ICU following cardiac surgery. We hypothesize that higher GPR values will be associated with increased risk of in-hospital mortality. Understanding this relationship could potentially identify a novel and easily obtainable marker to assist in the risk stratification of post-cardiac surgery ICU patients.

## 2. Methods

### 2.1. Study design and data source

This retrospective cohort study utilized data from the MIMIC-IV database (version 3.1), encompassing the period from 2008 to 2019.^[12]^ The study was conducted in compliance with ethical standards, and the need for informed consent was waived due to the use of de-identified data.

### 2.2. Patient selection

**Figure 1.**
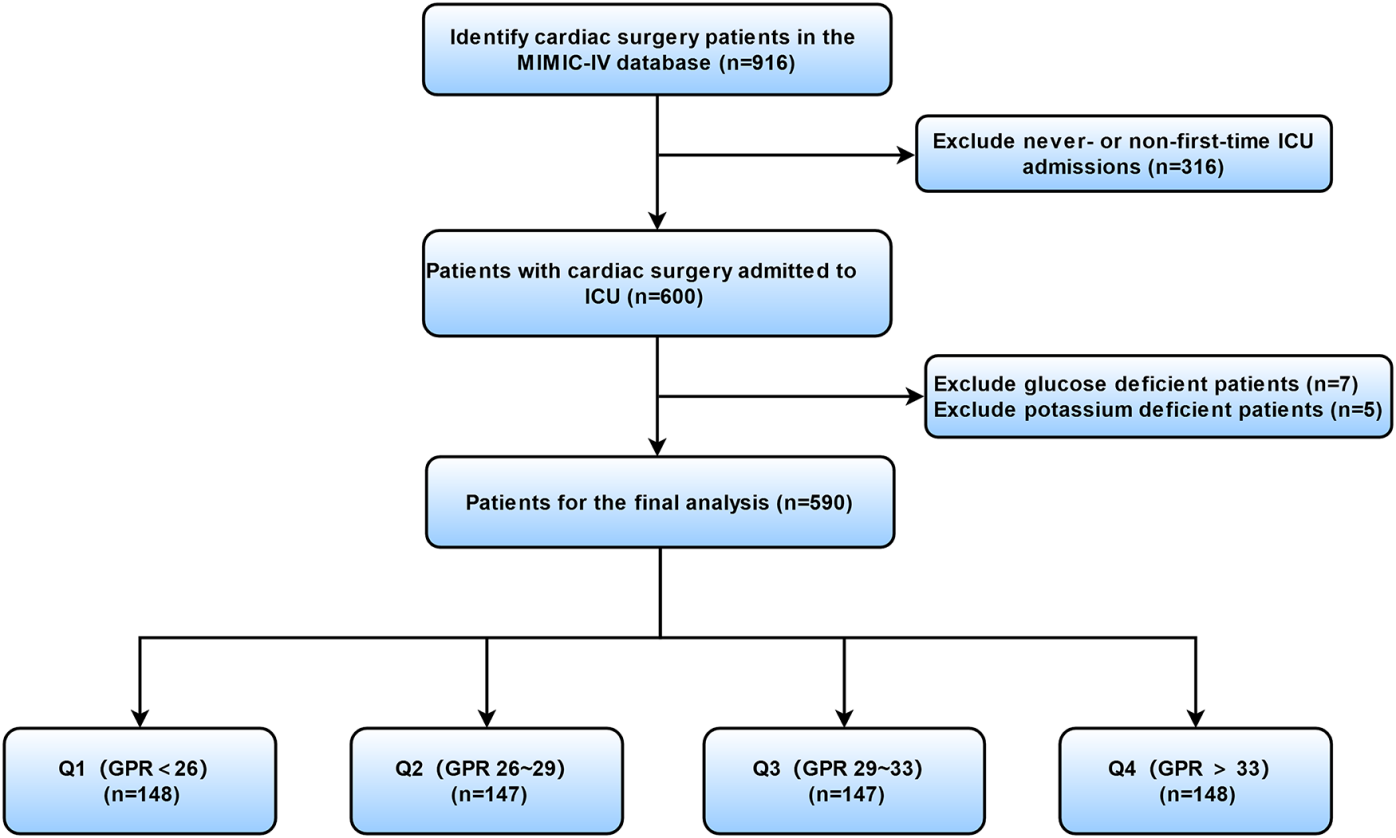
Flowchart of patient inclusion.

Patients undergoing cardiac surgery were identified using ICD codes: I97110, I97120, I97130, I97190, I97710, I97790, I97810, I97820, and 4294. The study included patients admitted to the ICU post-cardiac surgery. Exclusions were applied for non-first-time ICU admissions (n = 316), glucose-deficient patients (n = 7), and potassium-deficient patients (n = 5), resulting in a final cohort of 590 patients. The patient inclusion flowchart is depicted in Figure 1. The criteria for excluding non-first-time ICU admissions were based on the potential for confounding due to prior ICU stays, which could influence both GPR and mortality outcomes.

### 2.3. Data extraction and variables

Data retrieval for this study was carried out by a researcher who has completed the necessary training in human subject’s research and has been certified to access the database (certificate number 65828445). All relevant variables were documented within the first 24 hours following admission to the ICU. The key variable of interest in this study was the ratio of serum GPR. In addition to GPR, data collection encompassed patient demographics, existing health conditions, and various laboratory test results. The process of data extraction was guided by a predefined list of variables relevant to the study objectives, ensuring consistency and completeness.

### 2.4. Statistical analysis

For the statistical analysis, we utilized R Statistical Software (Version 4.2.2) along with the Free Statistics Analysis Platform (Version 2.3).^[13]^ To address missing data, we applied multiple imputation via chained equations. The primary analytical approach was the Cox proportional hazards model, which was used to assess the relationship between the GPR and in-hospital mortality. We began with a univariate Cox regression and progressed to multivariate models that included adjustments for factors such as age, gender, race, body mass index (BMI), and clinical scoring systems including Acute Physiology and Chronic Health Evaluation III, Simplified Acute Physiology Score II, Organ Dysfunction Assessment System, and Sequential Organ Failure Assessment (SOFA). The final model further controlled for conditions like heart failure, chronic pulmonary disease, diabetes, and renal disease. Additionally, subgroup analyses were performed to explore potential differences in the association between GPR and mortality across various patient characteristics, including age, gender, race, BMI, heart failure, chronic pulmonary disease, diabetes, and renal disease, with interaction *P*-values calculated to evaluate subgroup heterogeneity. The assumptions of the Cox proportional hazards model were tested using Schoenfeld residuals, and no significant violations were observed.

## 3. Results

### 3.1. Patient characteristics

The study population comprised 590 patients with a mean age of 71.4 years. The majority were male (66.4%) and non-White (69.8%). The mean GPR was 28.8, with quartiles defined as Q1 (<26), Q2 (26–29), Q3 (29–33), and Q4 (>33). Baseline characteristics are detailed in Table 1.

**Table 1:** Baseline characteristics of the study population.

Variables	Total (n = 590)	Q1 (n = 148)	Q2 (n = 147)	Q3 (n = 147)	Q4 (n = 148)	*P*-value
Age, mean ± SD	71.4 ± 10.8	72.3 ± 10.5	71.5 ± 10.8	69.4 ± 10.4	72.6 ± 11.2	.047
Gender, n (%)						.003
Female	198 (33.6)	58 (39.2)	35 (23.8)	43 (29.3)	62 (41.9)	
Male	392 (66.4)	90 (60.8)	112 (76.2)	104 (70.7)	86 (58.1)	
Race, n (%)						.454
White	178 (30.2)	44 (29.7)	45 (30.6)	38 (25.9)	51 (34.5)	
Non-White	412 (69.8)	104 (70.3)	102 (69.4)	109 (74.1)	97 (65.5)	
BMI, mean ± SD	29.4 ± 6.0	28.8 ± 5.8	29.1 ± 5.2	29.4 ± 6.1	30.3 ± 6.7	.143
Heart rate, mean ± SD	79.9 ± 11.3	78.2 ± 11.0	77.8 ± 10.6	81.6 ± 9.1	82.0 ± 13.6	<.001
SBP, mean ± SD	112.9 ± 12.0	113.6 ± 12.7	112.3 ± 10.0	111.6 ± 9.2	114.3 ± 14.9	.210
DBP, mean ± SD	59.1 ± 8.8	59.7 ± 10.1	58.0 ± 7.9	59.1 ± 7.8	59.6 ± 9.1	.310
Temperature, mean ± SD	36.7 ± 0.4	36.7 ± 0.3	36.6 ± 0.4	36.7 ± 0.4	36.8 ± 0.5	.037
SpO_2_, mean ± SD	97.3 ± 1.8	97.4 ± 1.7	97.4 ± 1.5	97.3 ± 1.5	97.0 ± 2.4	.192
Hematocrit, mean ± SD	28.5 ± 5.7	28.5 ± 5.8	29.0 ± 5.4	28.1 ± 5.2	28.5 ± 6.5	.591
Hemoglobin, mean ± SD	9.4 ± 1.9	9.3 ± 2.0	9.5 ± 1.8	9.3 ± 1.8	9.3 ± 2.2	.566
Platelets, mean ± SD	145.6 ± 66.6	145.8 ± 67.8	131.7 ± 51.7	143.1 ± 69.0	161.7 ± 73.0	.001
WBC, mean ± SD	16.3 ± 7.2	15.3 ± 9.2	16.0 ± 5.6	17.2 ± 6.6	16.6 ± 6.8	.129
Anion gap, mean ± SD	13.0 ± 4.0	12.6 ± 3.5	11.8 ± 3.3	12.4 ± 3.6	15.2 ± 4.8	<.001
Bicarbonate, mean ± SD	20.9 ± 3.1	21.7 ± 2.6	21.3 ± 2.5	20.8 ± 2.7	19.8 ± 3.9	<.001
Bun, mean ± SD	21.0 ± 11.7	23.6 ± 14.8	19.5 ± 9.7	18.4 ± 8.8	22.3 ± 11.8	<.001
Calcium, mean ± SD	8.3 ± 0.7	8.4 ± 0.7	8.2 ± 0.5	8.1 ± 0.5	8.3 ± 0.9	.004
Chloride, mean ± SD	103.5 ± 4.3	103.3 ± 4.5	104.1 ± 3.2	104.2 ± 4.0	102.6 ± 5.3	.004
Creatinine, median (IQR)	1.0 (0.8–1.3)	1.0 (0.8–1.5)	1.0 (0.8–1.2)	0.9 (0.8–1.2)	1.0 (0.9–1.4)	<.001
Sodium, mean ± SD	136.8 ± 3.5	136.9 ± 3.6	136.7 ± 2.5	136.9 ± 3.2	136.7 ± 4.3	.936
Potassium, mean ± SD	4.7 ± 0.6	5.0 ± 0.6	4.7 ± 0.4	4.6 ± 0.6	4.4 ± 0.5	<.001
Fibrinogen, mean ± SD	250.4 ± 105.4	253.1 ± 104.6	218.1 ± 66.1	233.0 ± 102.8	297.1 ± 123.3	<.001
INR, mean ± SD	1.5 ± 0.5	1.5 ± 0.4	1.5 ± 0.5	1.5 ± 0.4	1.5 ± 0.6	.751
PT, mean ± SD	16.7 ± 7.5	16.0 ± 4.0	16.6 ± 5.3	16.5 ± 4.1	17.5 ± 12.7	.367
APTT, median (IQR)	35.3 (29.7–54.8)	34.7 (29.2–54.0)	33.3 (29.4–42.7)	33.4 (29.1–48.4)	42.2 (31.2–79.4)	<.001
Lactate, median (IQR)	2.5 (1.8–3.6)	2.2 (1.6–3.0)	2.5 (2.0–3.2)	2.6 (1.9–3.5)	2.9 (1.9–4.6)	<.001
Glucose, mean ± SD	141.4 ± 37.8	117.5 ± 15.8	128.6 ± 10.8	139.4 ± 17.4	179.9 ± 53.1	<.001
APSIII, mean ± SD	42.0 ± 20.6	41.5 ± 20.6	38.1 ± 18.3	40.1 ± 20.5	48.5 ± 21.5	<.001
SAPSII, mean ± SD	37.9 ± 11.6	38.7 ± 12.1	37.3 ± 11.3	36.9 ± 11.0	38.5 ± 12.0	.457
OASIS, mean ± SD	31.8 ± 8.4	31.1 ± 8.2	31.0 ± 7.9	31.9 ± 8.5	33.2 ± 8.8	.081
SOFA, mean ± SD	5.3 ± 2.9	5.3 ± 3.4	5.2 ± 2.7	5.4 ± 2.7	5.2 ± 2.9	.859
Heart failure, n (%)						<.001
No	361 (61.2)	73 (49.3)	97 (66)	110 (74.8)	81 (54.7)	
Yes	229 (38.8)	75 (50.7)	50 (34)	37 (25.2)	67 (45.3)	
Chronic pulmonary, n (%)						.039
No	485 (82.2)	112 (75.7)	121 (82.3)	121 (82.3)	131 (88.5)	
Yes	105 (17.8)	36 (24.3)	26 (17.7)	26 (17.7)	17 (11.5)	
Diabetes, n (%)						<.001
No	437 (74.1)	132 (89.2)	122 (83)	109 (74.1)	74 (50)	
Yes	153 (25.9)	16 (10.8)	25 (17)	38 (25.9)	74 (50)	
Renal disease, n (%)						<.001
No	446 (75.6)	94 (63.5)	120 (81.6)	126 (85.7)	106 (71.6)	
Yes	144 (24.4)	54 (36.5)	27 (18.4)	21 (14.3)	42 (28.4)	

BUN = blood urea nitrogen, DBP = diastolic blood pressure, GPR = glucose–potassium ratio, INR = International Normalized Ratio, OASIS = Organ Dysfunction Assessment System, PT = prothrombin time, SAPS II = Simplified Acute Physiology Score II, SOFA = Sequential Organ Failure Assessment, SpO2 = Peripheral Capillary Oxygen Saturation, WBC = white blood cell.

### 3.2. Univariate cox regression analysis

The univariate Cox regression model showed a significant association between GPR and in-hospital mortality (hazard ratio [HR]: 1.06, 95% confidence interval [CI]: 1.03–1.08, *P* < .001). Other significant predictors included anion gap, bicarbonate, creatinine, potassium, fibrinogen, activated partial thromboplastin time, lactate, glucose, Acute Physiology and Chronic Health Evaluation III, Simplified Acute Physiology Score II, Organ Dysfunction Assessment System, and SOFA score (Table 2).

**Table 2:** A univariate Cox regression model evaluated the association between GPR and in-hospital mortality in patients with post-cardiac surgery.

Item	HR (95% CI)	*P*-value
Age (per 1 year)	1.03 (0.99–1.06)	.152
Sex (male vs female)	0.55 (0.27–1.1)	.091
Race (non-White vs White)	0.83 (0.41–1.7)	.616
BMI (per 1 kg/m^2^)	1.01 (0.97–1.06)	.570
Heart rate (per 1 beat/min)	1.0046 (0.9753–1.0349)	.760
Systolic BP (per 1 mm Hg)	0.99 (0.96–1.02)	.416
Diastolic BP (per 1 mm Hg)	0.9918 (0.9526–1.0325)	.687
Temperature (per 1 °C)	1.02 (0.49–2.09)	.961
SpO_2_ (per 1%)	0.95 (0.85–1.06)	.390
Hematocrit (per 1%)	1.03 (0.97–1.1)	.308
Hemoglobin (per 1 g/dL)	1.1 (0.91–1.32)	.339
Platelets (per 10^9^/L)	0.9966 (0.991–1.0021)	.225
WBC (per 10^9^/L)	1.02 (0.99–1.06)	.130
Anion gap (per 1 unit)	1.29 (1.21–1.37)	<.001
Bicarbonate (per 1 mmol/L)	0.79 (0.73–0.87)	<.001
Blood urea nitrogen (per 1 mg/dL)	1.0036 (0.9813–1.0264)	.756
Calcium (per 1 mg/dL)	1.52 (0.98–2.34)	.059
Chloride (per 1 mmol/L)	0.97 (0.91–1.04)	.389
Creatinine (per 1 mg/dL)	1.25 (1.1–1.42)	<.001
Sodium (per 1 mmol/L)	1.03 (0.94–1.14)	.500
Potassium (per 1 mmol/L)	1.79 (1.25–2.56)	.002
Fibrinogen (per 1 mg/dL)	1.0023 (1.0003–1.0043)	.026
INR (per 1 unit)	0.87 (0.42–1.83)	.720
PT (per 1 s)	0.99 (0.93–1.05)	.685
APTT (per 1 s)	1.02 (1.01–1.02)	<.001
Lactate (per 1 mmol/L)	1.25 (1.17–1.33)	<.001
Glucose (per 1 mmol/L)	1.02 (1.01–1.02)	<.001
APSIII (per 1 point)	1.02 (1.01–1.04)	<.001
SAPS II score (per 1 point)	1.05 (1.03–1.08)	<.001
OASIS (per 1 point)	1.07 (1.03–1.12)	.001
SOFA score (per 1 point)	1.08 (0.98–1.21)	.133
Heart failure (yes vs no)	2.53 (1.11–5.74)	.027
Chronic pulmonary disease (yes vs no)	0.66 (0.25–1.72)	.392
Diabetes mellitus (yes vs no)	0.88 (0.41–1.92)	.755
Renal_disease (yes vs no)	1.64 (0.8–3.33)	.175
GPR	1.06 (1.03–1.08)	<.001

APTT = activated partial thromboplastin time, CI = confidence interval, GPR = glucose–potassium ratio, HR = hazard ratio, SAPS II = Simplified Acute Physiology Score II, SOFA = Sequential Organ Failure Assessment, SpO2 = peripheral capillary oxygen saturation, WBC = white blood cell.

### 3.3. Multivariate cox regression analysis

In the multivariate analysis, GPR remained significantly associated with in-hospital mortality across all models (Model 3: HR: 1.07, 95% CI: 1.04–1.11, *P* < .001). Quartile analysis revealed a significant trend test (*P* = .040), with Q4 showing the highest risk of mortality compared to Q1 (Table 3).

**Table 3:** A multivariate Cox regression model evaluated the association between GPR and in-hospital mortality in patients with post-cardiac surgery.

Variable	No.	Crude model		Model 1		Model 2		Model 3	
		HR (95% CI)	*P*-value	HR (95% CI)	*P*-value	HR (95% CI)	*P*-value	HR (95% CI)	*P*-value
GPR	590	1.06 (1.03–1.08)	<.001	1.06 (1.04–1.09)	<.001	1.06 (1.03–1.09)	<.001	1.07 (1.04–1.11)	<.001
GPR (quartile)									
Q1 (<26)	148	1 (Ref)		1 (Ref)		1 (Ref)		1 (Ref)	
Q2 (26–29)	147	0.92 (0.27–3.2)	.902	0.93 (0.27–3.22)	.908	1.03 (0.29–3.59)	.966	0.98 (0.27–3.54)	.977
Q3 (29–33)	147	0.54 (0.14–2.08)	.369	0.54 (0.14–2.1)	.376	0.61 (0.15–2.45)	.487	0.7 (0.17–2.88)	.623
Q4 (>33)	148	2.52 (1.05–6.04)	.038	2.38 (0.98–5.8)	.056	2.51 (1–6.35)	.051	2.76 (1.05–7.29)	.040
Trend test	590	1.41 (1.03–1.93)	.033	1.37 (1–1.89)	.050	1.39 (1.01–1.92)	.045	1.43 (1.02–2)	.040

Crude model (unadjusted).

Model 1: adjusted for age, gender, race, and BMI.

Model 2: Model 1 plus APSIII score, SAPS II score, OASIS score, and SOFA score.

Model 3: Model 2 plus heart failure, chronic pulmonary disease, diabetes, and renal disease.

APSIII = Acute Physiology and Chronic Health Evaluation III, CI = confidence interval, GPR = glucose–potassium ratio, HR = hazard ratio, INR = International Normalized Ratio, OASIS = Organ Dysfunction Assessment System, SAPS II = Simplified Acute Physiology Score II, SOFA = Sequential Organ Failure Assessment.

### 3.4. Restricted cubic spline curve and survival analysis

**Figure 2.**
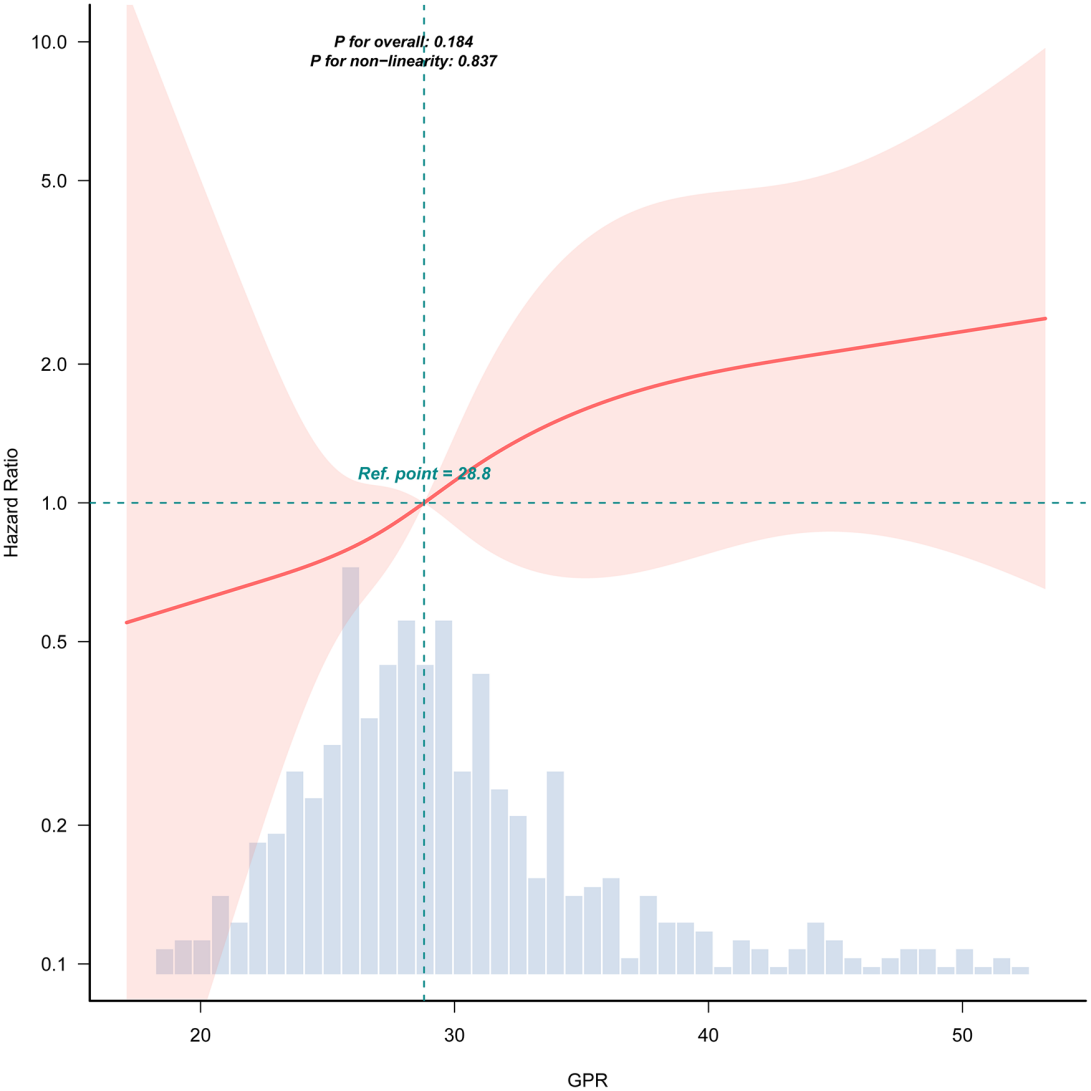
RCS curve for the GPR. GPR (quartile): Q1 (<26), Q2 (26–29), Q3 (29–33) , Q4 (>33). GPR = glucose–potassium ratio, RCS = restricted cubic spline.

**Figure 3.**
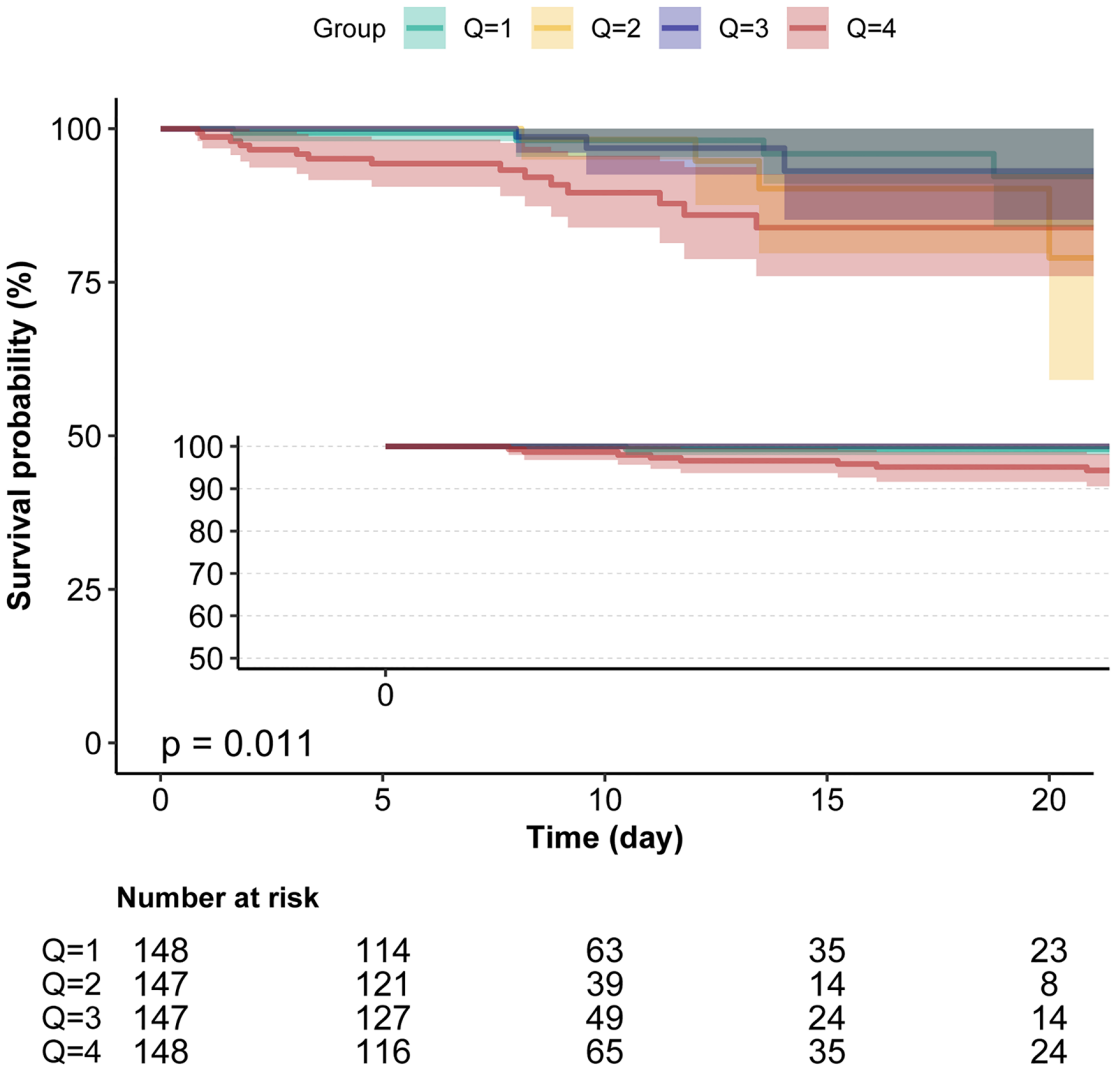
Kaplan–Meier survival analysis curves for in-hospital mortality.

The restricted cubic spline curve indicated a nonlinear relationship between GPR and HR, with a reference point at 28.8 (Fig. 2). Kaplan–Meier survival analysis showed significantly lower survival probabilities for patients in Q4 compared to other quartiles (*P* = .011) (Fig. 3).

### 3.5. Subgroup analysis

**Figure 4.**
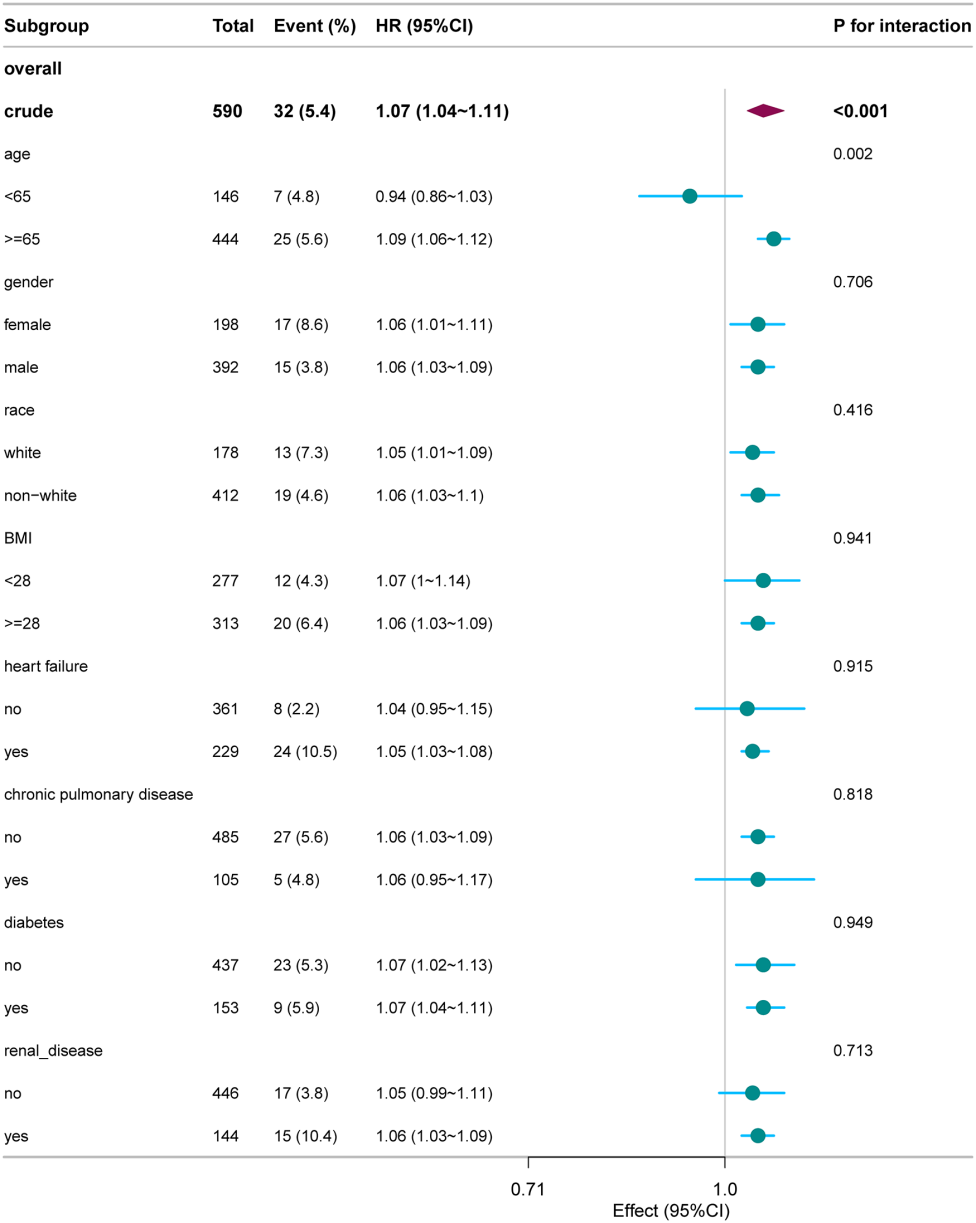
Forest plot for the subgroup analysis of the relationship between in-hospital mortality and GPR. GPR = glucose–potassium ratio.

Subgroup analyses revealed a significant interaction between GPR and age (*P* for interaction = 0.002), indicating effect modification by age. No other significant interactions were observed for the remaining prespecified subgroups (including gender, race, BMI, heart failure, chronic pulmonary disease, diabetes, and renal disease). The forest plot in Figure 4 illustrates the subgroup-specific HRs.

## 4. Discussion

This study’s findings indicate that a higher serum GPR is associated with an increased risk of in-hospital mortality among ICU patients following cardiac surgery. This relationship persists after adjusting for various confounders, suggesting that GPR may serve as a clinically relevant marker for risk stratification.

The association between GPR and mortality could be mediated through several biological pathways.^[10]^ Hyperglycemia, often observed in critically ill patients, can lead to increased oxidative stress, inflammation, and cellular dysfunction.^[8]^ These factors can exacerbate organ damage and impair wound healing, potentially leading to worse outcomes.^[4]^ Additionally, hyperglycemia has been linked to an increased risk of infection and sepsis, which are significant contributors to mortality in the ICU setting.^[14–16]^ Potassium imbalances can significantly impact cardiac function, with both hypokalemia and hyperkalemia leading to arrhythmias, which are known risk factors for mortality in critically ill patients.^[17]^ The balance of glucose and potassium is crucial for maintaining cellular homeostasis, and disruptions in this balance, as reflected by an elevated GPR, could indicate underlying metabolic stress.^[18,19]^

Previous studies have highlighted the prognostic value of individual components of GPR, such as hyperglycemia and potassium levels, in various clinical settings.^[20–24]^ For example, Charles et al demonstrated that intensive insulin therapy to maintain normoglycemia significantly reduced mortality in critically ill patients,^[25]^ while the importance of potassium balance in the ICU has also been well documented.^[26,27]^ However, fewer studies have examined the combined effect of glucose and potassium levels as reflected by GPR. Our findings align with those of Shan et al, who reported that GPR was associated with mortality in heart failure with preserved ejection fraction patients,^[28]^ suggesting its potential as a useful marker across different cardiovascular conditions. Our study extends these findings by specifically examining the post-cardiac surgery population, which faces unique metabolic challenges.

### 4.1. Limitations

Despite the intriguing findings, our study is not without limitations. The retrospective design of our analysis means that we cannot infer causality from the observed associations. The potential for unmeasured confounding factors that could influence both GPR and mortality outcomes exists. For instance, the timing and frequency of glucose and potassium measurements could vary, potentially affecting the accuracy of GPR as a marker. The generalizability of our results may be limited due to the specific population studied. The patients in theICU MIMIC-IV database are predominantly from a single-center setting, which may not be representative of all ICU populations worldwide. Additionally, the database does not provide information on long-term outcomes, such as readmission rates or quality of life, which are important considerations in the overall assessment of patient care. Furthermore, the use of multiple imputation for missing data introduces uncertainty. While multiple imputation is a widely accepted method for handling missing data, it relies on assumptions about the missing data mechanism, which may not always be met. Lastly, the timing of GPR measurement within 24 hours of ICU admission may not capture the dynamic changes in glucose and potassium levels that could occur over the course of hospitalization. In this regard, a more significant limitation is our reliance on a single, static GPR value. While our findings demonstrate an association between an initial elevated GPR and mortality, a single measurement cannot reflect a patient’s clinical trajectory. A declining GPR might indicate metabolic stabilization and recovery, whereas a rising or persistently elevated GPR could signal clinical deterioration or poor response to therapy. Therefore, the absence of serial GPR measurements (i.e., a GPR trend) limits our ability to use this marker for monitoring improvement or worsening of the clinical condition over time, and future studies should incorporate longitudinal assessments.

Furthermore, our findings reveal a significant interaction between GPR and age, as well as a strong association with renal function, which have direct clinical implications that must be acknowledged as limitations of our passive observational approach. Regarding age (significant interaction), older patients may have blunted counter-regulatory responses or polypharmacy effects that alter both glucose and potassium handling. Consequently, any management approach targeting GPR would need to be age-stratified. Regarding renal function (association without interaction), although no statistical interaction was found, the strong association between GPR and renal function as a potential confounder remains. An elevated GPR may be influenced by reduced potassium excretion, a common feature of acute kidney injury or chronic kidney disease. We did not adjust for baseline estimated glomerular filtration rate or urine output, nor did we analyze GPR according to different stages of renal impairment. Therefore, we cannot propose a specific management strategy (e.g., aggressive glucose control vs potassium modulation) without risking harm. For example, attempting to lower GPR by administering insulin would reduce potassium further, potentially precipitating life-threatening hypokalemia, especially in older patients with tenuous electrolyte balance. Given these complexities, any management approach targeting GPR would need to be age- and renal function-stratified, with careful avoidance of potassium correction without concurrent glucose monitoring. Our study was not designed to evaluate such interventions, and until causal mechanisms are clarified, GPR should remain a risk stratification tool rather than a therapeutic target.

### 4.2. Future research

Future studies should aim to validate our findings in different patient populations to assess the generalizability of GPR as a prognostic marker. However, a key question is whether large, expensive randomized controlled trials are immediately necessary, or if a more incremental approach is warranted. Given the observational nature of our study and the unresolved issues of confounding and causation, we propose a stepped research agenda. First, focused retrospective reviews from different centers (with diverse case mixes and practice patterns) should be prioritized. Such multi-center retrospective analyses would help determine whether the GPR–mortality association is robust across settings or is highly context-dependent. If consistent effect sizes and interactions (particularly with age) are observed across multiple datasets, this would strengthen the rationale for prospective evaluation. Second, and more importantly, a prospective cohort study should be conducted to test the marker’s predictive performance in real time. This would involve measuring GPR serially (e.g., daily for the first 5–7 ICU days) and assessing its ability to predict not only mortality but also other patient-centered outcomes (e.g., new renal replacement therapy, arrhythmias, length of stay). Only if such prospective testing demonstrates incremental prognostic value beyond existing clinical scores (SOFA, APACHE) would a pilot interventional trial be justified: for example, a before-after study evaluating whether a GPR-directed electrolyte and glucose management protocol improves outcomes. Randomizing patients to a GPR-targeted intervention would be premature at this stage. Therefore, we do not yet need large randomized controlled trials; rather, the priority is to replicate our findings in multi-center retrospective datasets and then validate them prospectively before any interventional studies are considered.

## 5. Conclusion

In conclusion, our analysis of the MIMIC-IV database suggests that GPR is associated with in-hospital mortality in patients following cardiac surgery. While this finding is promising, it should be interpreted with caution given the study’s limitations. Further research is needed to confirm the prognostic value of GPR and to explore its potential role in clinical practice. Understanding the mechanisms through which GPR influences outcomes could provide valuable insights into the pathophysiology of critical illness and may guide the development of targeted interventions.

## Author contributions

**Conceptualization:** Yan Wu, Guang Tu.

**Data curation:** Yan Wu, Dandan Xu, Jun Lu, Lin Zhang, Zhonglan Cai, Guang Tu.

**Formal analysis:** Dandan Xu, Jun Lu, Lin Zhang, Zhonglan Cai.

**Funding acquisition:** Guang Tu.

**Investigation:** Yan Wu, Dandan Xu, Jun Lu, Lin Zhang, Zhonglan Cai, Guang Tu.

**Methodology:** Dandan Xu, Jun Lu, Lin Zhang, Zhonglan Cai.

**Project administration:** Yan Wu, Guang Tu.

**Supervision:** Yan Wu, Guang Tu.

**Writing – original draft:** Yan Wu, Dandan Xu, Jun Lu, Lin Zhang, Zhonglan Cai, Guang Tu.

**Writing – review & editing:** Yan Wu, Guang Tu.
